# Structural lubricity under ambient conditions

**DOI:** 10.1038/ncomms12055

**Published:** 2016-06-28

**Authors:** Ebru Cihan, Semran İpek, Engin Durgun, Mehmet Z. Baykara

**Affiliations:** 1 UNAM—Institute of Materials Science and Nanotechnology, Bilkent University, Ankara 06800, Turkey; 2 Department of Mechanical Engineering, Bilkent University, Ankara 06800, Turkey

## Abstract

Despite its fundamental importance, physical mechanisms that govern friction are poorly understood. While a state of ultra-low friction, termed structural lubricity, is expected for any clean, atomically flat interface consisting of two different materials with incommensurate structures, some associated predictions could only be quantitatively confirmed under ultra-high vacuum (UHV) conditions so far. Here, we report structurally lubric sliding under ambient conditions at mesoscopic (∼4,000–130,000 nm^2^) interfaces formed by gold islands on graphite. *Ab initio* calculations reveal that the gold–graphite interface is expected to remain largely free from contaminant molecules, leading to structurally lubric sliding. The experiments reported here demonstrate the potential for practical lubrication schemes for micro- and nano-electromechanical systems, which would mainly rely on an atomic-scale structural mismatch between the slider and substrate components, via the utilization of material systems featuring clean, atomically flat interfaces under ambient conditions.

## Abstract (web summary)

Structural lubricity—referring to ultralow levels of friction between atomically flat, incommensurate surfaces—has previously been observed under ultrahigh vacuum. Here, the authors report structural lubricity at gold-graphite interfaces under ambient conditions and on mesoscopic scales.

## Graphical abstract

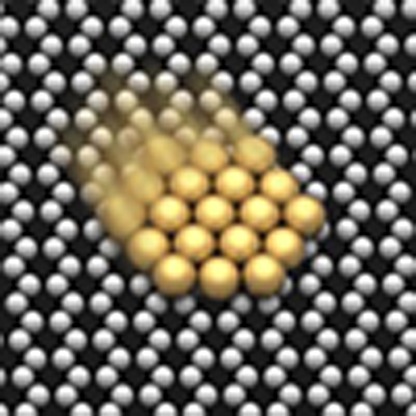

Friction is a ubiquitous phenomenon encountered during every-day activities as common as walking, and also holds primary importance in mechanical processes as the main mechanism responsible for energy dissipation^1^. Moreover, due to large surface-to-volume ratios associated with components featured in micro- and nano-electromechanical systems, friction constitutes major limits to efficient and reliable operation of such devices^2^.

Scientific efforts directed towards gaining a fundamental understanding of friction have accelerated over the last few decades, primarily due to the development of the atomic force microscope (AFM)^3^. In particular, friction forces measured at the single-asperity represented by the AFM probe tip have been thoroughly investigated as a function of load, contact size, sliding speed and temperature^4^. On the other hand, issues involving limited control over contact area, poorly characterized tip structures and limited choice of materials for AFM cantilevers have led to the development of lateral manipulation experiments for friction research^5^.

The phenomenon of structural lubricity (also referred to as superlubricity^6^, see Supplementary Note 1) is of fundamental importance in friction. Specifically, any rigid interface formed by two atomically flat, incommensurate surfaces that is free from contaminant molecules is expected to undergo sliding with ultra-low friction, characterized by a sub-linear relationship between friction force and contact area^7^,^8^,^9^,^10^. Despite the simplicity of the underlying physical principle, structurally lubric sliding between different materials in quantitative agreement with a sub-linear scaling law has only been confirmed under ultra-high vacuum (UHV) conditions so far^11^. The absence of reports regarding structurally lubric sliding between arbitrary combinations of atomically flat surfaces under ambient conditions has been primarily attributed to the presence of mobile contaminant molecules adjusting to potential energy minima at the interface^12^. On the other hand, certain experiments have revealed that sliding with ultra-low friction under non-vacuum conditions can be achieved between the individual, atomically flat layers of carbon-based materials such as double-walled carbon nanotubes^13^ and graphite^14^,^15^,^16^, as well as a material system consisting of graphene, diamond-like carbon and nanoscale diamond particles^17^. In addition, under vacuum conditions, there have been reports of ultra-low friction sliding at very small contacts formed by scanning probe microscopy tips^18^ and the absence of static friction for adsorbed monolayers of, for example, Kr on gold surfaces as measured by quartz crystal microbalance experiments^19^.

Here, we perform AFM-based lateral manipulation experiments under ambient conditions on gold islands situated on graphite (see the ‘Methods' section)^20^, to study the dependence of friction force on contact area at the interface formed between the two materials, and to probe the potential occurrence of structural lubricity. Results reveal that gold islands exhibiting atomically flat contact areas of ∼4,000–130,000 nm^2^ with the graphite substrate experience ultra-low friction forces (<2.5 nN) during sliding. In addition, a study of the dependence of friction force on contact area leads to the determination of sub-linear scaling factors, in agreement with the theory of structural lubricity. The discovery that structural lubricity at mesoscopic interfaces consisting of surfaces formed by two different materials may be achieved under ambient conditions paves the way to the development of practical structural lubrication schemes for micro- and nano-electromechanical systems.

## Results

### Structure of gold islands on graphite

**Figure 1:**
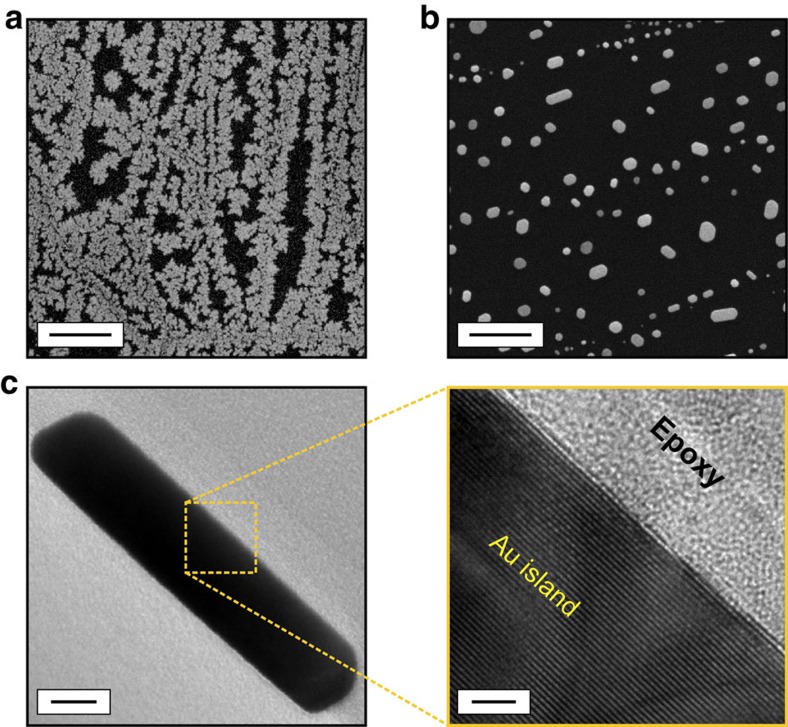
Structural characterization of gold islands on graphite. (**a**) A representative SEM image of the thin film formed on graphite after thermal deposition of 1 Å gold. Scale bar, 500 nm. (**b**) An SEM image of the graphite surface decorated with gold islands of various size after post-deposition annealing at 650 °C. Scale bar, 500 nm. (**c**) Cross-sectional TEM images of an individual gold island. The highlighted high-resolution image confirms the crystalline structure of the gold island, as well as the absence of an oxide layer. Scale bars, 10 nm and 2 nm, respectively.

Thermal evaporation of 1 Å gold on graphite results in the presence of a thin film with sub-monolayer coverage (Fig. 1a). Post-deposition annealing leads to the formation of well-faceted gold islands with a wide distribution of lateral size (Fig. 1b). While the predominantly straight facets exhibited by the gold islands are indicative of crystalline structure, we have performed transmission electron microscopy (TEM) experiments to directly confirm the crystalline character of the islands (Fig. 1c). High-resolution, cross-sectional TEM images reveal the crystalline order of the gold islands, with (111) planes oriented parallel to the graphite substrate. In addition, in contrast to antimony islands investigated in the past via manipulation experiments under ambient conditions^21^, the absence of an oxide layer on the gold island surfaces is observed. This observation is in alignment with previous work that shows gold, which is known to be of extremely inert character^22^, only demonstrates chemical reactivity in nano particle form at a size regime that is significantly smaller than the islands used in our study (typically below 10 nm)^23^.

### Lateral manipulation of gold islands on graphite

To perform lateral manipulation experiments, AFM has been utilized in contact mode^24^. To limit the magnitude of forces exerted on the sample, soft silicon cantilevers have been used (see the ‘Methods' section). Despite the use of soft cantilevers and the low magnitude of normal forces (<1 nN), AFM experiments resulted in the lateral manipulation of the majority of gold islands during scanning, while a smaller number of islands trapped at/between the step edges of graphite or other surface defects remained—at least, temporarily—stationary.

**Figure 2:**
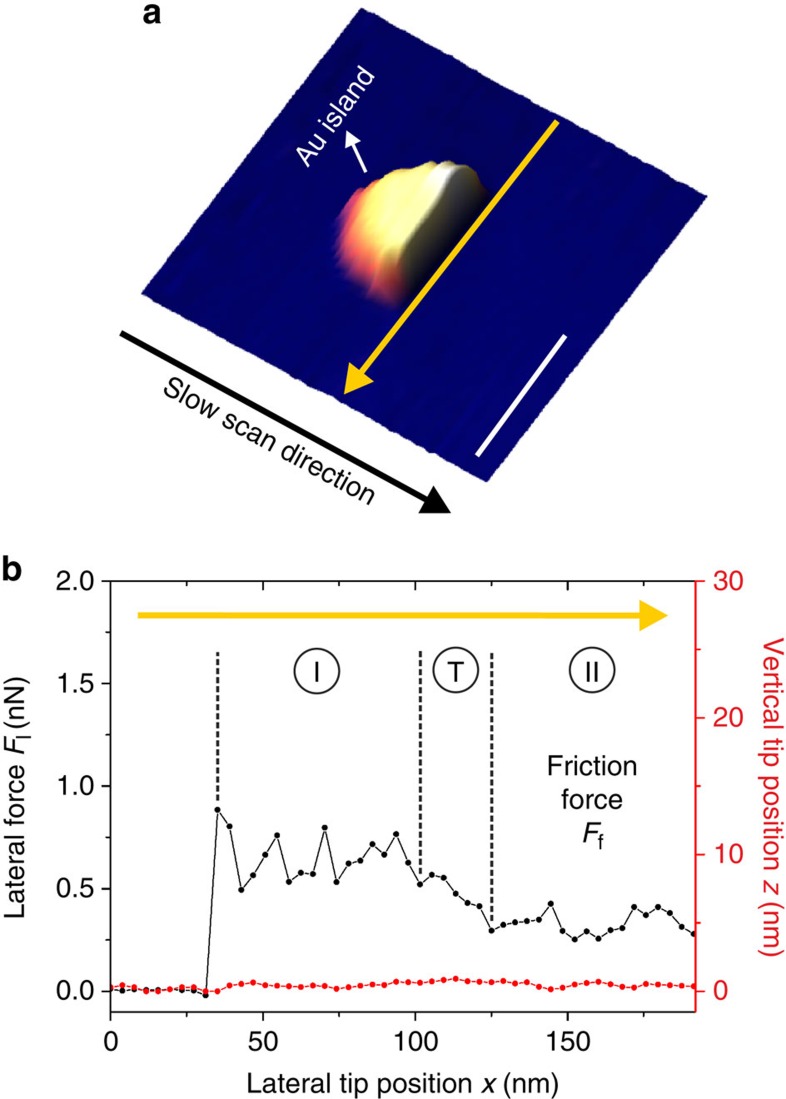
Lateral manipulation of gold islands on graphite. (**a**) Three-dimensional representation of an AFM image detailing the lateral manipulation of a single gold island on graphite. The island is manipulated by the AFM tip along the yellow arrow, and thus appears ‘cut' afterwards. Scale bar, 100 nm. (**b**) The lateral force *F*_l_ (black) and vertical tip position *z* (red) signals recorded during manipulation along the yellow arrow. Note that the *z* signal remains constant during manipulation, thus confirming that the tip pushes the island from the side. While recorded *F*_l_ values consistently and remarkably remain below 1 nN, two regions of relatively high and low friction (denoted by ‘I' and ‘II') can be discerned, separated by a short transition regime (denoted by ‘T'). The interfacial friction value *F*_f_ and associated error bars are extracted from region II.

On the basis of the observation that gold islands are readily manipulated by the AFM tip during scanning, indicative of low frictional resistance to motion, we have directed our efforts at quantifying the related friction forces during manipulation. A representative manipulation event is demonstrated in Fig. 2a, where a gold island is laterally pushed by the AFM tip along the yellow arrow. An investigation of vertical tip position (*z*) and lateral force (*F*_l_) signals recorded along the manipulation line reveals that an increase in *F*_l_ is recorded during manipulation, corresponding to interfacial friction between the island and the substrate (Fig. 2b). Remarkably, the recorded values of *F*_l_ remain below 1 nN during the entire scan line, in very good agreement with manipulation experiments performed on gold islands on graphite under UHV^11^, and about three orders of magnitude smaller than the results reported for antimony islands under ambient conditions^25^ (for a discussion regarding the effect of humidity on graphite in the context of our experiments, see Supplementary Note 2). In addition, two regions of relatively high and low friction can be observed. In the initial phase of the manipulation (region I), the lateral force signal remains relatively high (0.65±0.11 nN), while lateral force values eventually drop to a lower value (0.33±0.05 nN) after a short transition regime (region II). It should be indicated that interfacial friction force values (*F*_f_) for each manipulation event in our studies are extracted from region II of sliding, where such a distinction can be made (see the ‘Methods' section).

### Dependence of friction force on contact area

**Figure 3:**
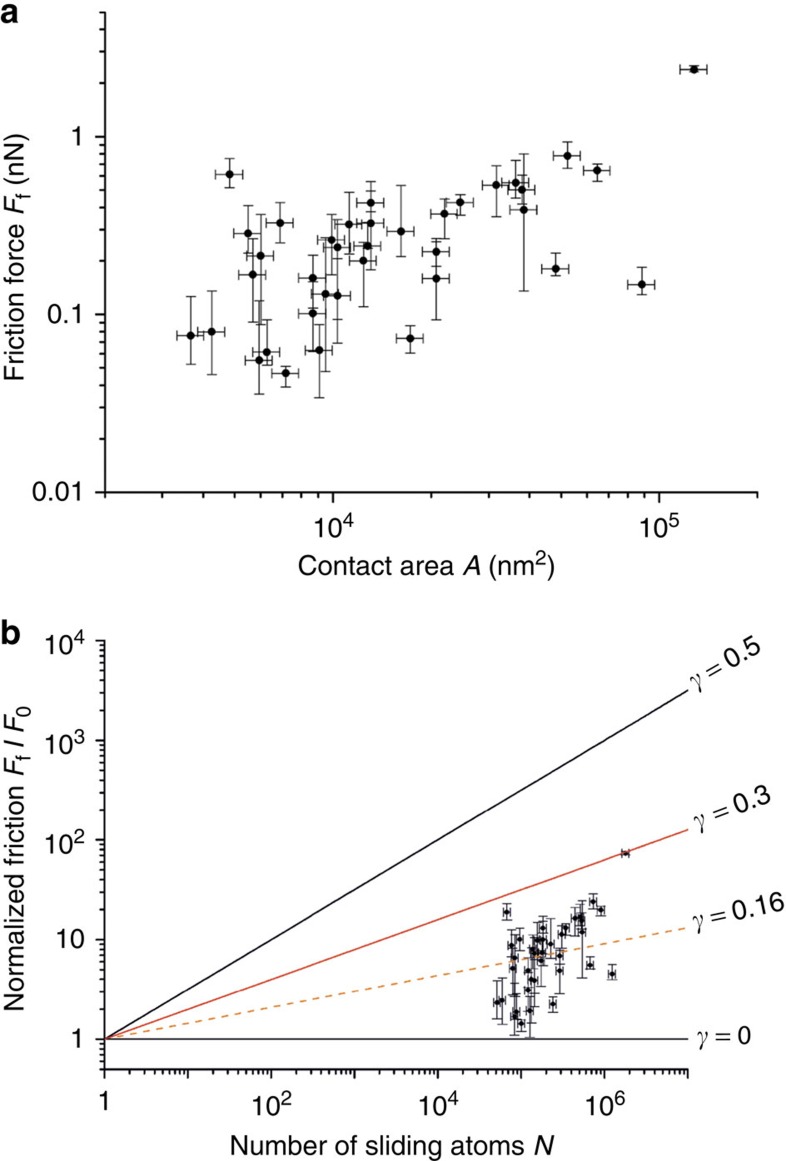
Structural lubricity under ambient conditions. (**a**) Interfacial friction force (*F*_f_) values for 37 manipulation events plotted as a function of contact area *A*. (**b**) Normalized friction force values (*F*_f_/*F*_0_) plotted as a function of number of atoms on the sliding gold surface *N*. All manipulation events fall clearly (*γ*≤0.3) within the regime defined for structural lubricity (0<*γ*<0.5), with a mean scaling power of *γ*=0.16. The relatively large spread in the data is attributed to the variability in the circumferential shape of the gold islands, with some islands exhibiting more straight facets and sharper corners than others^11^. Horizontal error bars of ±10% are imposed on *A* and *N* values due to tip-convolution effects^44^. Vertical error bars associated with *F*_f_ and *F*_f_/*F*_0_ values are directly extracted from individual manipulation events as in Fig. 2b.

To quantitatively confirm the occurrence of structurally lubric sliding, *F*_f_ shall be investigated as a function of interfacial contact area *A*. Toward this purpose, manipulation experiments have been performed on a number of gold islands exhibiting contact areas of ∼4,000–130,000 nm^2^ with the graphite substrate. The theory of structural lubricity predicts for crystalline interfaces that *F*_f_ shall scale sub-linearly with *A* (ref. 10), and consequently, the number of atoms on the sliding surface *N*, such that:


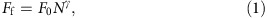


where, *F*_0_ is the ‘theoretical friction force' expected for a single atom sliding on the substrate, as determined by the ratio of the related diffusion-energy barrier, Δ*E*, and the lattice constant, *a*. *γ* is the scaling power and is expected to be between 0 and 0.5, depending on the shape as well as the relative orientation of the slider with respect to the substrate^10^,^11^. For a gold slider manipulated over graphite, Δ*E*=50 meV (ref. 26) (Supplementary Note 3 and Supplementary Fig. 1) and *a*=0.246 nm. *N* can be determined from *A* by considering the density of atoms on the (111) surface of gold, *ρ*_Au_=14.03 atoms nm^−2^. The dependence of *F*_f_ on *A* is plotted in Fig. 3a, for 37 manipulation events. Friction values remain outstandingly low for all manipulated islands, with the maximum amount of friction force (2.38 nN) experienced by the largest island (∼130,000 nm^2^). To validate the occurrence of sub-linear evolution of friction with respect to contact area in accordance with equation (1), normalized friction values for each manipulation event (*F*_f_/*F*_0_) are plotted as a function of *N* in Fig. 3b. All manipulation events clearly fall within the range defined for structurally lubric sliding (0<*γ*<0.5). It should be indicated that the results presented in Fig. 3 are in striking similarity to the results obtained via manipulation of gold islands under UHV conditions on graphite^11^, such that there is a considerable quantitative overlap between the friction force values observed for similarly sized islands under both experimental conditions.

## Discussion

**Figure 4:**
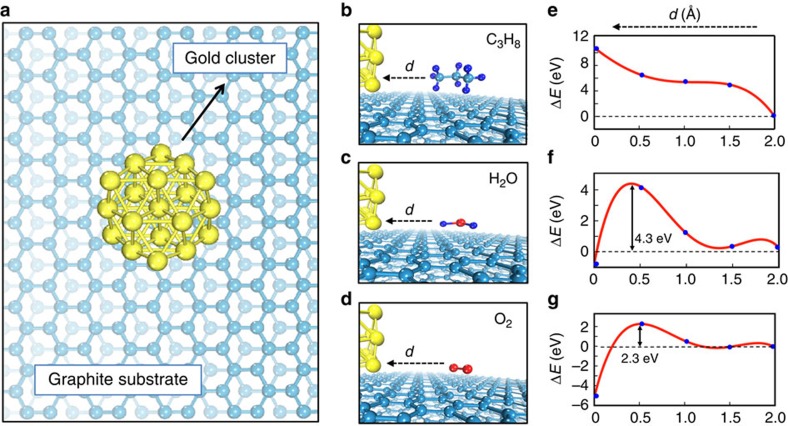
***Ab initio*** **simulations** **of the interaction between the gold–graphite interface and contaminant molecules.** (**a**) A top-view illustration of the model system used for the calculations, consisting of a 19-atom gold cluster on a 3-layer graphite substrate. (**b**–**d**) Side-view illustrations of propane (**b**), water (**c**) and oxygen (**d**) molecules approaching the gold–graphite interface with decreasing distance *d*. (**e**–**g**) Calculated change in the total energy of the system (Δ*E*) as a function of *d* for the three scenarios in **b**–**d**. While the propane molecule is repelled by the interface due to a steeply increasing energy penalty (**e**), substantial energy barriers of 4.3 eV (**f**) and 2.3 eV (**g**) are observed for the dissociation of water and oxygen at the interface, respectively. For improved visualization, the quadratic spline interpolation method was utilized in **e**–**g**.

While the consistent observation of structurally lubric sliding between gold islands and graphite under ambient conditions is remarkable, the results appear to be in contradiction with the argument that mobile contaminant molecules in the sliding interface between two atomically flat surfaces lead to the breakdown of structural lubricity^12^, a prediction that has been partially verified via comparative manipulation experiments performed on antimony islands under UHV and ambient conditions^25^. A similar mechanism has also been suggested to result in the breakdown of structurally lubric behaviour of MoS_2_, when introduced from UHV to ambient conditions^27^. To investigate the interaction between the gold–graphite interface and common contaminant molecules, *ab initio* simulations based on density functional theory (DFT) have been performed (see the ‘Methods' section). Toward this purpose, a 19-atom gold cluster consisting of 3 layers of gold atoms configured in (111) planes was situated on a 3-layer graphite substrate consisting of 153 carbon atoms (Fig. 4a), resulting in a calculated spacing of 3.45 Å between the gold cluster and the graphite surface. Single molecules of propane (a representative hydrocarbon-based contaminant), water and oxygen were approached to the gold–graphite interface in steps of 0.5 Å (Fig. 4b–d) to obtain minimum-energy paths, and the resulting evolution in the total energy of the system (Δ*E*) was calculated (Fig. 4e–g). The results reveal that propane experiences a steeply increasing repulsive interaction with decreasing distance *d*, and is consequently repelled by the gold–graphite interface (Fig. 4e). While water and oxygen also undergo repulsive interactions with decreasing *d*, both molecules can be dissociated if brought sufficiently close to the interface, requiring energy barriers of 4.3 and 2.3 eV to do so, respectively (Fig. 4f,g). Both energy barriers are sufficiently high, such that dissociation and subsequent adsorption at the gold–graphite interface are not expected at room temperature. Consequently, we expect the atomically flat gold–graphite interfaces investigated in our experiments to remain largely free from contaminant molecules including hydrocarbons, water and oxygen; which would in turn lead to the occurrence of structurally lubric sliding. In fact, the robustness of the gold–graphite interface with respect to the contaminant molecules would be expected to result in the observation of similar friction force values for similarly sized gold islands under both UHV^11^ and ambient conditions, which is exactly the case in the experiments presented here.

As the occurrence of structural lubricity at the interface between two atomically flat, crystalline surfaces with incommensurate lattice structures is theoretically expected regardless of the chemical identity of the atoms forming the surfaces (Supplementary Note 4 and Supplementary Fig. 2), a natural question would be whether the observations reported here are unique to the specific material system investigated (gold islands on graphite) or whether it is possible to achieve structural lubricity on graphite under ambient conditions with islands made of other elements, for example, antimony or copper. While AFM-based manipulation experiments on nano-/meso-scale islands are quite scarce, previous results obtained via manipulation of antimony islands under ambient conditions have revealed a mostly linear (that is, not structurally lubric) friction force versus contact area relationship with friction forces that are orders of magnitude larger than those reported here for gold islands^21^,^25^ (Supplementary Note 4 and Supplementary Fig. 3). As the main physical difference between the gold islands investigated in our work, and the antimony islands investigated in the reported efforts in the context of surface structure is the existence of an amorphous oxide layer on the antimony islands (Supplementary Fig. 4), it becomes evident that the existence of an amorphous oxide layer results in a breakdown of structural lubricity. In fact, theoretical studies have revealed (i) the possibility for the existence of mobile antimony oxide asperities as a potential source of increased friction under ambient conditions for such islands^28^, and (ii) that the atomic-scale roughness of amorphous sliders has a dominant effect on friction^12^,^29^,^30^. Thus, the chemical inertness of the gold islands used in our experiments and the associated absence of an amorphous oxide layer emerge as critical factors leading to structurally lubric sliding under ambient conditions. The robustness of gold islands studied in the experiments reported here against oxidation under ambient conditions opens up remarkable possibilities for practical applications of the idea of structural lubricity as a viable lubrication scheme for micro- and nano-electromechanical systems, with components under relative sliding motion that involve, for example, high-efficiency mechanical actuation with minimal friction and wear. On the other hand, further experiments aimed towards the characterization of the conservation of structural lubricity with respect to (i) increasing contact size, and (ii) sliding history are needed to fully realize the discussed potential.

## Methods

### Sample preparation

Samples were prepared by a two-step process: (i) Thermal deposition of 999.9-purity gold on highly oriented pyrolytic graphite substrates (ZYB-quality, Ted Pella) and (ii) post-deposition annealing of the gold-coated graphite substrates in a quartz-tube furnace (Alser Teknik/ProTherm) or a rapid thermal annealing instrument (ATV Technologie) at 650 °C for 1–2 h. Graphite substrates were prepared by cleaving in air via adhesive tape, followed by immediate transfer to the vacuum chamber of the thermal evaporator (Vaksis). Evaporation took place at a base pressure of 5 × 10^−6^ Torr, and at a deposition rate of 0.1 Å s^−1^ for a typical total deposited amount of 1 Å, with the graphite substrate held at room temperature. Post-deposition annealing led to the formation of gold islands in (elongated)-hexagonal shapes with predominantly straight facets, with lateral sizes up to ∼500 nm.

### Structural characterization via SEM and TEM

Samples were structurally characterized via scanning electron microscopy (SEM; FEI Quanta 200 FEG, typically operated at 10 kV) to investigate the size and distribution of gold islands on the graphite substrate. No special treatment was necessary for SEM imaging of the as-prepared samples. To confirm the crystalline character, TEM was utilized (FEI Tecnai G2 F30, typically operated at 300 kV). The TEM samples were prepared in two different ways: (1) To investigate individual gold islands via regular (top-view) TEM imaging, a thin layer of the gold-covered graphite sample was mechanically peeled off and subsequently sonicated in ethanol, which was followed by drop-casting on a Cu grid (300 mesh). (2) For cross-sectional TEM imaging, samples were prepared via focused ion beam milling (FEI Nova 600 Nanolab). Initially, a region of the sample containing several gold islands was coated with epoxy to protect the surface during ion milling and then, a thin lamella was carved via ion beam. The cut lamella was tilted for pre-thinning; the final, fine thinning was applied at low ion beam energies. Finally, the resulting sample was placed on the TEM grid via Pt-welding in the focused ion beam instrument for subsequent cross-sectional imaging.

### Lateral manipulation experiments via AFM

A commercial AFM instrument (PSIA XE-100E) was utilized in contact mode to perform the lateral manipulation experiments on the gold islands situated on graphite, in accordance with well-established procedures in the literature^24^. Soft cantilevers designed for contact mode imaging (Nanosensors PPP-CONTR series, radius of curvature *r*≅10 nm) were used during the experiments, and calibration of related normal- and lateral-stiffness values was performed via the methods reported by Sader *et al.*^31^ and Varenberg *et al.*^32^, respectively. Typical normal spring constant values (*k*) were 0.20 N m^−1^, and typical lateral force calibration factors (*α*) were 15 nN V^−1^. All presented data have been obtained at low applied normal loads (<1 nN) and under relative humidity values of 20–30%. As the majority of gold islands were spontaneously manipulated by the AFM tip during scanning (even at vanishingly small applied normal loads), and the associated investigation of interfacial friction was hard to perform practically in most cases, we have primarily focused on islands that were initially immobile at a step edge or another defect on the graphite surface, but were eventually manipulated by the AFM tip during repeated scanning of the respective surface region. The occurrence of relatively high lateral force values in region I of sliding in comparison with region II is likely caused by this fact. On the other hand, based on the observation that the extent of region I is on a similar scale to the lateral size of the gold island itself in Fig. 2b, it may be argued that gold islands experience lower friction values on fresh areas of graphite when compared with the locations which they are initially situated on. Nevertheless, a thorough clarification of this aspect is beyond our current experimental capabilities. Contact areas were determined via topographical AFM images^24^, taking into account that the gold islands expose atomically flat surfaces on the graphite substrate. Scanning and, consequently, manipulation was performed at a typical speed of 1 μm s^−1^. Lateral force values were collected with a density of 1 data point per ∼10 nm in a typical scan line—as such, the potential occurrence of stick-slip motion during particle motion or the effect of time-dependent rotational switching to pseudo-commensurate configurations during sliding^33^ cannot be investigated in our data sets. Finally, a potential breakdown of structurally lubric sliding at high loads^34^ cannot be probed in our experiments, as the manipulation was performed by the AFM tip pushing the islands from the side rather than the alternative tip-on-top mode^35^, which allows control over normal loads acting at the interface between the islands and the substrate.

### *Ab initio* calculations

The calculations were performed by first-principles computational techniques based on DFT^36^,^37^ implemented in the Vienna *ab initio* simulation package (VASP)^38^,^39^. The exchange-correlation potential was approximated within the generalized gradient approximation including van der Waals correction according to the DFT+D2 approach^40^, which has been previously shown to deliver accurate results for the interaction of gold with hydrocarbons^41^. We used projector-augmented-wave potentials^42^, and the exchange-correlation potential was described by the Perdew–Burke–Ernzerhof functional^43^. The calculations were done at Γ-point, using a plane-wave basis set with a kinetic-energy cutoff of 500 eV. All structures were optimized with simultaneous minimization of the total energy and inter-atomic forces. The convergence on the total energy and force was set to 10^−5^ eV and 10^−2^ eV Å^−1^, respectively. A symmetry constraint was imposed on gold clusters to preserve the hexagonal symmetry at small sizes. Single molecules of propane, oxygen and water were approached to the gold–graphite interface in steps of 0.5 Å to obtain minimum-energy paths, and the resulting evolution in the total energy of the system was calculated. Obtained results were further tested with larger systems consisting of five-layered gold clusters and graphite substrates, which yielded similar results.

### Data availability

The data that support the findings of this study are available from the corresponding author on request.

## Additional information

**How to cite this article:** Cihan, E. *et al.* Structural lubricity under ambient conditions. *Nat. Commun.* 7:12055 doi: 10.1038/ncomms12055 (2016).

## Supplementary Material

Supplementary Information Supplementary Figures 1-4, Supplementary Notes 1-4 and Supplementary References
