# Supplementary material for: Structural lubricity under ambient conditions
Source: Nat Commun. 2016 Jun 28;7:12055. doi: 10.1038/ncomms12055 (PMC4931278; doi:10.1038/ncomms12055)
Supplement: Supplementary Information — Supplementary Figures 1-4, Supplementary Notes 1-4 and Supplementary References [file ncomms12055-s1.pdf]

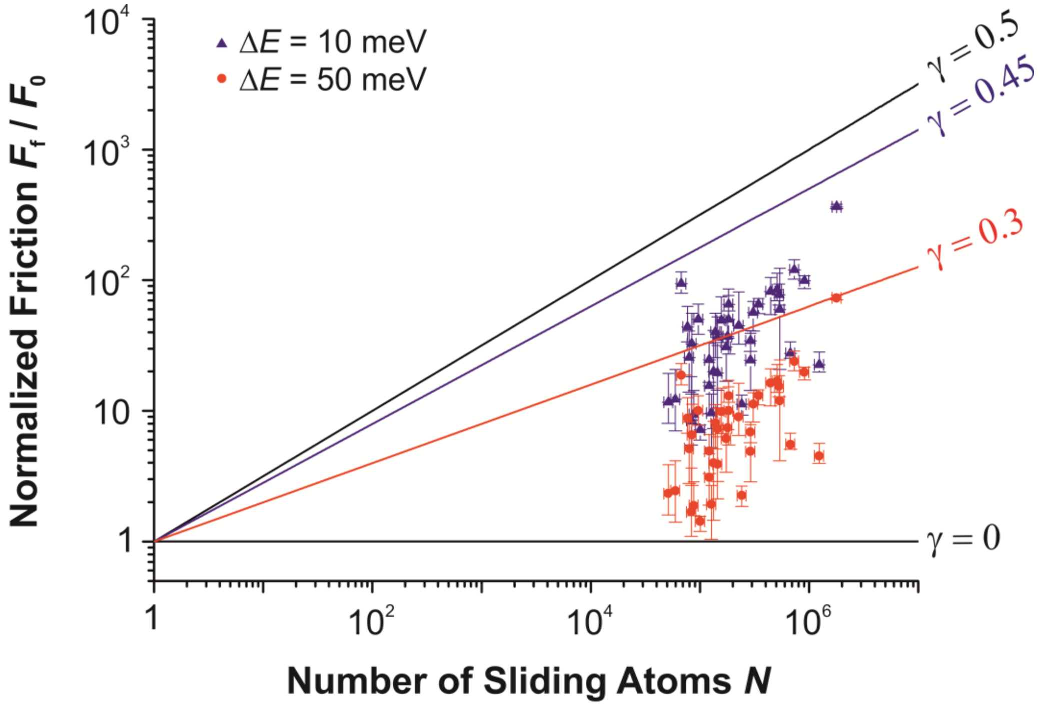

Supplementary Figure 1 | Structural lubricity under ambient conditions for different diffusion energy barriers. Normalized friction force values (F_f/F_0) plotted as a function of number of atoms on the sliding gold surface (N) for two different diffusion energy barriers (ΔE) of 10 meV (violet triangles) and 50 meV (red circles). For an underestimated ΔE value of 10 meV, the experimental data still remain in the regime defined for structural lubricity ($0 < \gamma < 0.5$), with an upper bound on the scaling power corresponding to $\gamma = 0.45$.

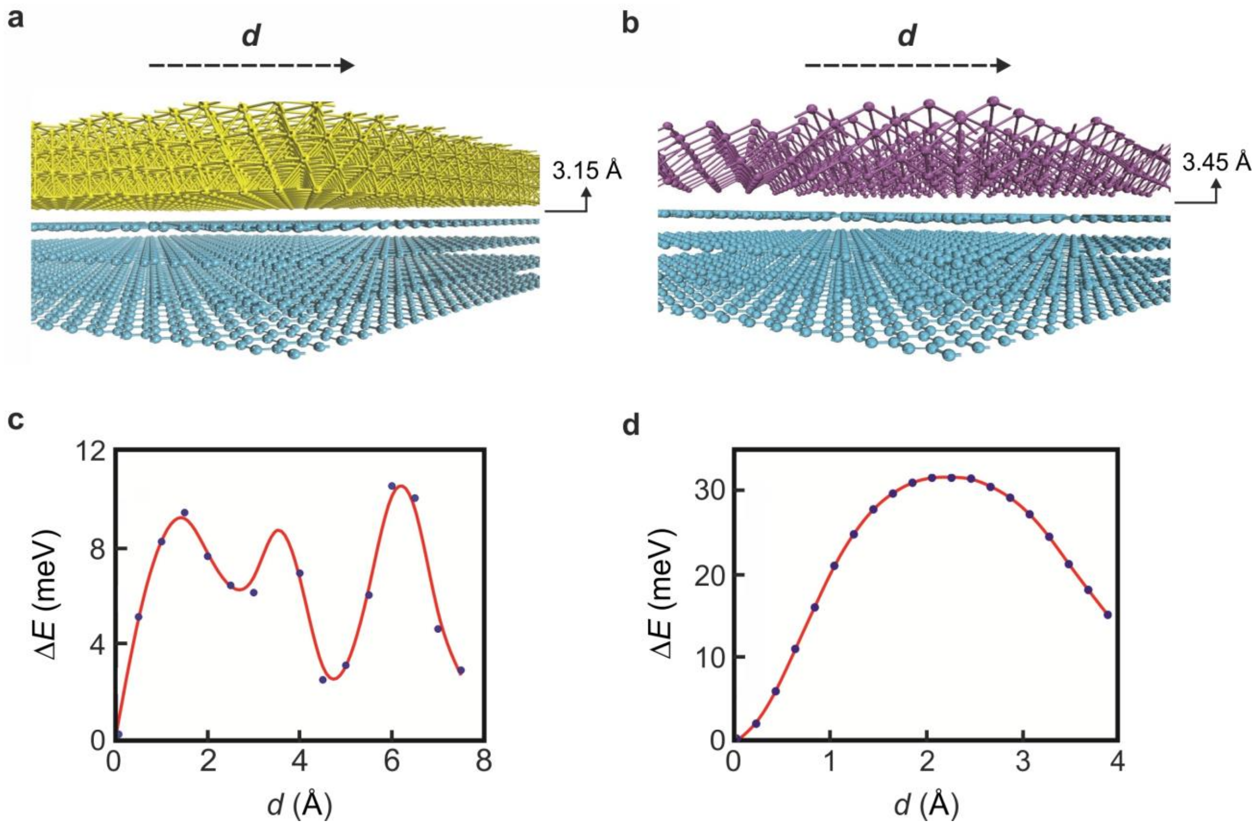

Supplementary Figure 2 | *Ab initio* simulations of sliding for gold-graphite and antimony-graphite interfaces. (a) Side view of the model gold-graphite system used for calculations. (**b)** Side view of the model antimony-graphite system used for calculations. The small spacing between the slabs and the substrate (3.15 Å and 3.45 Å, respectively) are indicated. **(c-d)** Calculated change in the total energy of the system (ΔE) as a function of sliding distance (d) for the scenarios in (a-b). The more *regular* evolution of ΔE vs. d for the antimony slab when compared to gold is due to the fact that it is at a “nearly commensurate” configuration with the underlying substrate. For improved visualization, the quadratic spline interpolation method was utilized in (c-d).

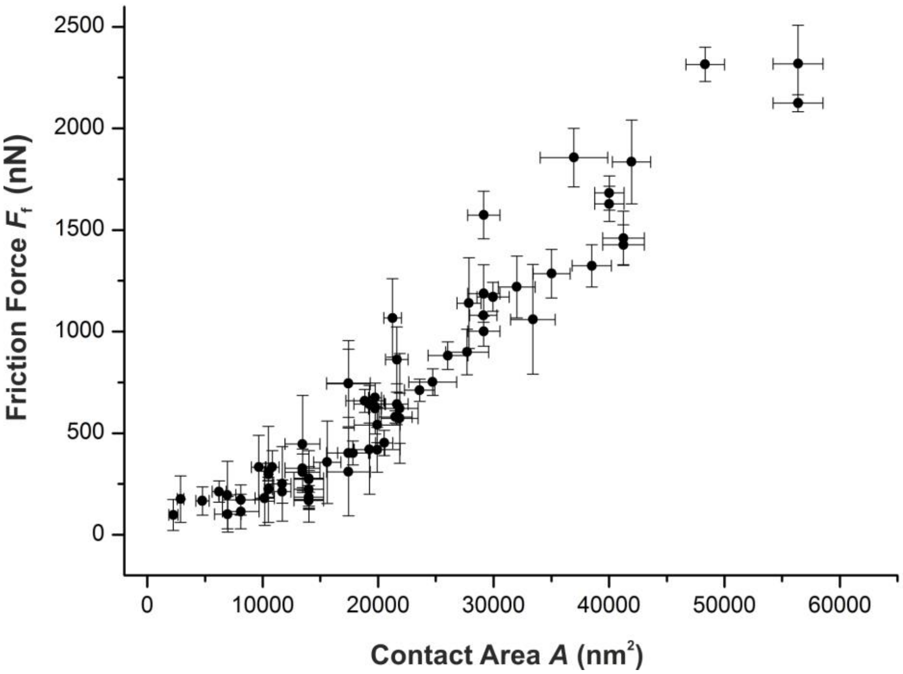

Supplementary Figure 3 | Dependence of friction force on contact area for antimony islands sliding on graphite under ambient conditions. Friction forces experienced by antimony islands sliding on graphite under ambient conditions are orders of magnitude larger than those experienced by gold islands. The smallest antimony island ($A \approx 2250$ nm^2) experiences a friction force of ~100 nN. Adapted from Ref. [1]. Copyright 2013 by the American Physical Society.

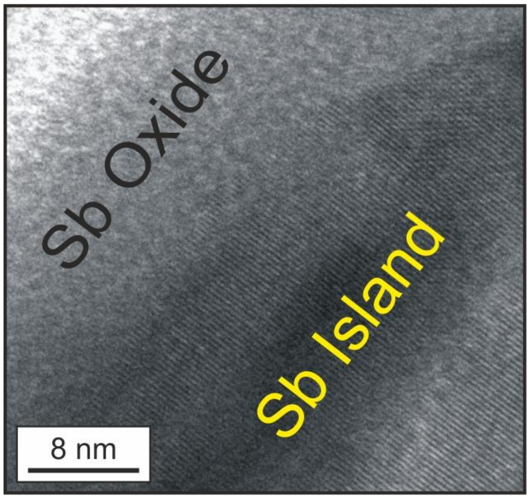

Supplementary Figure 4 | High-resolution, cross-sectional TEM image of an individual antimony island. The high-resolution image reveals that the crystalline bulk of the antimony island is surrounded by an amorphous antimony oxide layer, leading to the breakdown of structural lubricity during sliding. Adapted from Ref. [1]. Copyright 2013 by the American Physical Society.

Supplementary Note 1: Structural lubricity vs. superlubricity

In nanotribology literature, the expressions *structural lubricity* and *superlubricity* are often used interchangeably, which typically leads to certain confusion regarding the associated physical mechanisms as well as misleading comparisons to the phenomenon of superconductivity[2]. While the term *superlubricity* may refer to any state of ultra-low friction sliding regardless of the underlying physical principles, *structural lubricity* deals with a state of ultra-low friction sliding arising specifically due to structural incommensurability associated with atomically flat surfaces in contact[3]. On the other hand, ultra-low friction (*i.e.*, superlubric) sliding may be obtained by alternative methods including (i) the reduction of the interfacial load below a threshold value during AFM-based friction experiments to enter a quasi-static sliding regime[4], (ii) the deliberate mechanical excitation of the tip-sample contact during AFM experiments[5,6], and (iii) increased temperatures leading to the onset of the *thermolubric* regime[7], characterized by the disappearance of *stick-slip* behavior and substantial reduction in friction.

Supplementary Note 2: Graphite and humidity

It has been known for multiple decades that the macroscopically observed lubricative properties of graphite improve significantly with increasing humidity[8,9]. On the other hand, the drastic improvement in the lubricative properties of graphite upon exposure to water molecules in macroscopic experiments has not been reproduced consistently for nano-scale experiments, performed, e.g., via AFM: In their seminal paper, Schwarz *et al.* have compared friction coefficients for HOPG under dry Argon and under ambient conditions, and have found little difference (0.008±0.005 *vs.* 0.005±0.003)[10]. This result suggests that for nano-scale experiments, the effect of water on the lubricative properties of graphite is not always well-defined.

Moreover, for manipulation experiments performed on nanoscale antimony islands on graphite under ambient conditions[1,11], the reported values of friction have always been significantly above the regime defined for structural lubricity. As such, it has been established that performing nano-manipulation experiments on graphite under ambient conditions does *not* automatically result in sliding with low friction due to *lubrication* by water; a suitable material system such as gold islands on graphite discussed here is indeed required for superlubricity. Finally, the close similarity between the friction force values we report under ambient conditions when compared to the UHV results of Dietzel *et al.*[12] readily indicates that the presence of water is not responsible for our results – a fact that is also supported by the *ab initio* calculations presented in our work, pointing to the robustness of the gold-graphite interface with respect to contaminant molecules including water.

Supplementary Note 3: Diffusion energy barrier for gold on graphite

The diffusion energy barrier for individual gold atoms on graphite (ΔE) has been previously determined to be 50 meV via *ab initio* calculations[13], which, when combined with the spacing of hollow sites on graphite (a = 0.246 nm), leads to a theoretical value of F_0 = 33 pN. As the value of F_0 (the *friction force* expected for a single gold atom sliding on graphite) is of critical importance for the quantitative validation of structurally lubric sliding in our experiments, we have taken the potential occurrence of a substantial over-estimation in the value of ΔE into account and have re-drawn Fig. 3b with an assumed diffusion barrier value of only 10 meV (Supplementary Fig. 1). While such a low diffusion energy barrier for the material system in question is unlikely, the corresponding results nevertheless confirm that the experimental data are still in the regime defined for structurally lubric sliding.

Supplementary Note 4: Breakdown of structural lubricity due to oxidation

The basic theory behind structural lubricity predicts that sliding at any clean interface consisting of atomically-flat, crystalline surfaces with different lattice structures should result in ultra-low friction values that scale sub-linearly with the number of sliding atoms, regardless of the particular chemical identity of the atoms making up the surfaces.

To re-confirm the validity of this idea, we have performed *ab initio* simulations of slabs of gold and antimony (consisting of 4 layers oriented in (111) configuration) sliding on a 4-layer graphite substrate in structurally incommensurate fashion (Supplementary Figure 2a,b). Antimony was chosen as a test material due to the availability of experimental data regarding its frictional behavior[1,11]. To test a *limiting case* for antimony, the slab was rotated only slightly away from the commensurate configuration with the underlying graphite substrate such that a "nearly commensurate" alignment is achieved. The gold and antimony slabs were slid along the dashed arrows displayed in Supplementary Fig. 2 (at zero temperature) and the resulting differences in energy were calculated as a function of sliding distance *d* (Supplementary Figure 2c,d). Subsequently, the friction forces that would be experienced by the sliding slabs were approximated by considering the steepest slope of the energy landscape in the direction of sliding that needs to be surmounted. Results reveal that the gold slab exposing 28 atoms to the graphite surface is predicted to be subjected to a friction force of ~11 pN. With the experimentally obtained scaling factor of 0.16, this result translates to a friction force of ~41 pN for a gold slider with 10^5 sliding atoms, within the range of our experimental findings, thus confirming the validity of the computational approach employed here. On the other hand, the antimony slab exposing 26 atoms to the graphite surface is expected to be subject to a friction force of ~23 pN. The same order of magnitude friction forces calculated for gold and antimony

sliders forming clean, incommensurate interfaces with graphite (despite the “nearly commensurate” alignment of the antimony slab with the graphite substrate) provides further proof that the occurrence of structural lubricity for atomically-flat, crystalline slider-substrate configurations should indeed be material-independent.

Based on the discussion above, the question whether structural lubricity under ambient conditions could be *experimentally* observed for nano/meso-scale islands of materials other than gold becomes an intriguing one. Despite the considerably small number of results reported in the literature regarding lateral manipulation experiments in general, a certain amount of data are available for the frictional behavior of antimony islands on graphite[1,11]. In particular, lateral manipulation experiments performed on antimony islands under ambient conditions invariably result in the observation of substantial friction forces (orders of magnitude above those expected for structurally lubric sliding) and a mostly linear dependence of friction force on contact area (Supplementary Figure 3).

As both experiments on antimony (reported before) and gold nanoparticles (investigated in the present work) are conducted under typical laboratory conditions using essentially the same experimental approach, the vast difference in frictional behavior must arise from physical differences of the surfaces that they expose to the graphite substrate: While the gold islands investigated in the present work do not feature any oxide layers at their surfaces (see Fig. 1c in the main text), it is observed that antimony readily oxidizes under ambient conditions and forms an amorphous antimony oxide layer around the islands (Supplementary Figure 4). Combined with the theoretical and computational work which demonstrate that atomic-scale roughness of amorphous sliders is expected to lead to a breakdown of structural lubricity and dominate frictional behavior[14,15], it is reasonable to conclude that the absence of oxidation (which is the

case for gold islands investigated in this work) is a prerequisite for the conservation of structural lubricity under ambient conditions.

Supplementary References

1. Ritter, C., Baykara, M. Z., Stegemann, B., Heyde, M., Rademann, K., Schroers, J. & Schwarz, U. D. Nonuniform friction-area dependency for antimony oxide surfaces sliding on graphite. *Phys. Rev. B* **88,** 045422 (2013).
2. Meyer, E. & Gnecco, E. Superlubricity on the nanometer scale. *Friction* **2,** 106-113 (2014).
3. Müser, M. H. Structural lubricity: Role of dimension and symmetry. *Europhys. Lett.* **66,** 97-103 (2004).
4. Socoliuc, A., Bennewitz, R., Gnecco, E. & Meyer, E. Transition from stick-slip to continuous sliding in atomic friction: Entering a new regime of ultralow friction. *Phys. Rev. Lett.* **92,** 134301 (2004).
5. Socoliuc, A. *et al.* Atomic-scale control of friction by actuation of nanometer-sized contacts. *Science* **313,** 207-210 (2006).
6. Lantz, M. A., Wiesmann, D. & Gotsmann, B. Dynamic superlubricity and the elimination of wear on the nanoscale. *Nature Nanotech.* **4,** 586-591 (2009).
7. Krylov, S. Y., Jinesh, K. B., Valk, H., Dienwiebel, M. & Frenken, J. W. M. Thermally induced suppression of friction at the atomic scale. *Phys. Rev. E* **71,** 065101 (2005).
8. Savage, R. H. Graphite lubrication. *J. Appl. Phys.* **19,** 1-11 (1948).
9. Bowden, F. P. & Young, J. E. Friction of diamond, graphite, and carbon and the influence of surface films. *Proc. R. Soc. A* **208,** 444-455 (1951).
10. Schwarz, U. D., Zwörner, O., Köster, P. & Wiesendanger, R. Quantitative analysis of the frictional properties of solid materials at low loads. I. Carbon Compounds *Phys. Rev. B* **56,** 6987-6996 (1997).

11. Dietzel, D. *et al.* Frictional duality observed during nanoparticle sliding. *Phys. Rev. Lett.* **101,** 125505 (2008).
12. Dietzel, D., Feldmann, M., Schwarz, U. D., Fuchs, H. & Schirmeisen, A. Scaling laws of structural lubricity. *Phys. Rev. Lett.* **111,** 235502 (2013).
13. Jensen, P., Blase, X. & Ordejon, P. First principles study of gold adsorption and diffusion on graphite. *Surf. Sci.* **564,** 173-178 (2004).
14. He, G., Müser, M. H. & Robbins, M. O. Adsorbed layers and the origin of static friction. *Science* **284,** 1650-1652 (1999).
15. Mo, Y. F., Turner, K. T. & Szlufarska, I. Friction laws at the nanoscale. *Nature* **457,** 1116-1119 (2009).
